# In situ-forming collagen hydrogel crosslinked via multi-functional PEG as a matrix therapy for corneal defects

**DOI:** 10.1038/s41598-020-72978-5

**Published:** 2020-10-07

**Authors:** Gabriella Maria Fernandes-Cunha, Karen Mei Chen, Fang Chen, Peter Le, Ju Hee Han, Leela Ann Mahajan, Hyun Jong Lee, Kyung Sun Na, David Myung

**Affiliations:** 1 Ophthalmology, Byers Eye Institute, Stanford University School of Medicine, Palo Alto, CA USA; 2 Chemical and Biological Engineering, Gachon University, Seongnam-si, Gyeonggi-do Republic of Korea; 3 Ophthalmology and Visual Science, Yeouido St. Mary’s Hospital, College of Medicine, The Catholic University of Korea, Seoul, Republic of Korea; 4 Chemical Engineering, Stanford University, Stanford, CA USA; 5 VA Palo Alto HealthCare System, Palo Alto, CA USA

**Keywords:** Biomaterials, Tissue engineering

## Abstract

Visually significant corneal injuries and subsequent scarring collectively represent a major global human health challenge, affecting millions of people worldwide. Unfortunately, less than 2% of patients who could benefit from a sight-restoring corneal transplant have access to cadaveric donor corneal tissue. Thus, there is a critical need for new ways to repair corneal defects that drive proper epithelialization and stromal remodeling of the wounded area without the need for cadeveric donor corneas. Emerging therapies to replace the need for donor corneas include pre-formed biosynthetic buttons and in situ-forming matrices that strive to achieve the transparency, biocompatibility, patient comfort, and biointegration that is possible with native tissue. Herein, we report on the development of an in situ-forming hydrogel of collagen type I crosslinked via multi-functional polyethylene glycol (PEG)-N-hydroxysuccinimide (NHS) and characterize its biophysical properties and regenerative capacity both in vitro and in vivo. The hydrogels form under ambient conditions within minutes upon mixing without the need for an external catalyst or trigger such as light or heat, and their transparency, degradability, and stiffness are modulated as a function of number of PEG arms and concentration of PEG. In addition, in situ-forming PEG-collagen hydrogels support the migration and proliferation of corneal epithelial and stromal cells on their surface. In vivo studies in which the hydrogels were formed in situ over stromal keratectomy wounds without sutures showed that they supported multi-layered surface epithelialization. Overall, the in situ forming PEG-collagen hydrogels exhibited physical and biological properties desirable for a corneal stromal defect wound repair matrix that could be applied without the need for sutures or an external trigger such as a catalyst or light energy.

## Introduction

Injuries and diseases of the cornea that lead to stromal defects are significant causes of disability, morbidity and vision loss worldwide^1,2^. In cases of corneal thinning or melt situations where perforation is imminent, off-label use of cyanoacrylate is employed to stabilize the cornea prior to a definitive but invasive corneal transplant^3^. However, cyanoacrylate glue creates an opaque, rough surface that provides no visual or regenerative benefits^4^. Penetrating and lamellar keratoplasty are invasive procedures and the waiting lists for human corneal transplantation include more than 10 million people globally^5^. Numerous efforts to create pre-formed biosynthetic donor corneal buttons using collagen, gelatin, and hyaluronic acid have been made^2,6–10^. Recently, Holoclar, a fibrin-based disc with stem cells was approved for use throughout the European Union to restore vision in patients with physical injuries and chemical burns^11^.

Recently, matrix therapies of various kinds have been developed^2–4,8,12,13^ where a flowable material is applied and cured over corneal wounds. The goal of a matrix therapy is to sustain epithelial and stromal cell ingrowth^4^, and specific priorities are transparency and biocompatibility to improve host-graft interaction^6,14^. In one study, dopamine hydrazone-crosslinked hyaluronic acid hydrogels were developed to enhance adhesivity to the corneal tissue and deliver limbal cells^8^. Samarawickrama et al. showed that collagen-like peptide conjugated to PEG-maleimide was created to seal acute corneal perforations while promoting tissue regeneration^3^. Collagen is a particularly attractive biomaterial for ocular adhesives because it is the most abundant protein in mammals, is naturally present in corneal stroma and is the main structural protein in the extracellular environment^15^. However, for collagen to be used in situ on a wounded corneal surface, the cytotoxicity of the chemistry being used to crosslink is a critical consideration^3,9,12,16,17^.

Recently, both photo-initiated and light-free in situ-forming hydrogel approaches have been shown to have promise in the repair of corneal stromal defects. Sani et al. demonstrated that a photocrosslinked methacryloyl-gelatin gel exhibited adhesive properties and supported surface epithelialization^18^. Li et al. reported on a similar photocurable gelatin-based method where acrylated gelatin is reacted with thiolated gelatin^13^. While these strategies do not require sutures, they do necessitate a photoinitiator and light energy for gelation. Although photochemistry has certain potential advantages and is used clinically and is FDA-approved for corneal crosslinking (CXL) via riboflavin, it also has known detrimental effects on stromal keratocytes and corneal nerves^19^. We recently reported on an in situ-forming collagen hydrogel that is crosslinked by bio-orthogonal copper-free click chemistry without the need for external triggers such as light, heat, or any chemical initiators, and demonstrated that it can support a multi-layered epithelium in corneal organ culture^12^.

Here, we present a light-free, in situ-forming approach to crosslinking collagen using multi-arm PEG using N-hydroxysuccinimide (NHS) chemistry to fill and repair corneal defects. This chemical crosslinking approach has been used clinically in sealants to stop fluid leaks in both neurological and ophthalmic surgery^20^. For instance, the FDA-approved product ReSure (Ocular Therapeutix, Bedford, MA) is a sealant for corneal incisions which utilizes an (NHS)-terminated 4-arm PEG prepolymer that reacts with the primary amines of trilysine to facilitate in situ gelation^21^. In other work, collagen has been crosslinked via multifunctional PEG to form gels that have been shown to be biocompatible and support cell growth in vitro^17^. Given its track record as a crosslinking chemistry for an ophthalmic sealant for corneal incisions^4^, we have utilized the succinimide active ester reaction to facilitate crosslinking of collagen in situ as a matrix therapy for corneal epithelial and stromal defect repair. The experiments reported herein were used to investigate the importance of PEG arm number and concentration on the material’s biophysical properties both in vitro and in vivo, and demonstrate the potential of multi-arm PEG-collagen hydrogels as in situ-forming, suture-free, and catalyst-free scaffolds to promote epithelial wound healing in the treatment of corneal defects.

## Results

### Mechanical properties of PEG-collagen hydrogels

**Figure 1:**
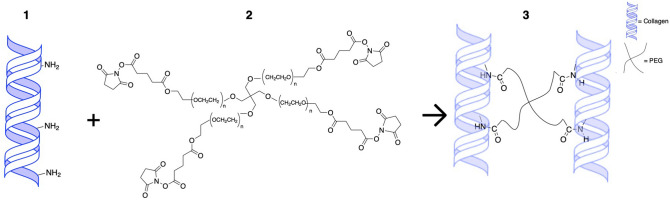
Schematic of the crosslinking between primary amines on collagen and NHS groups on multi-arm PEG polymers; (1) collagen, (2) 4-arm PEG-NHS (3) PEG-collagen hydrogel.

**Figure 2:**
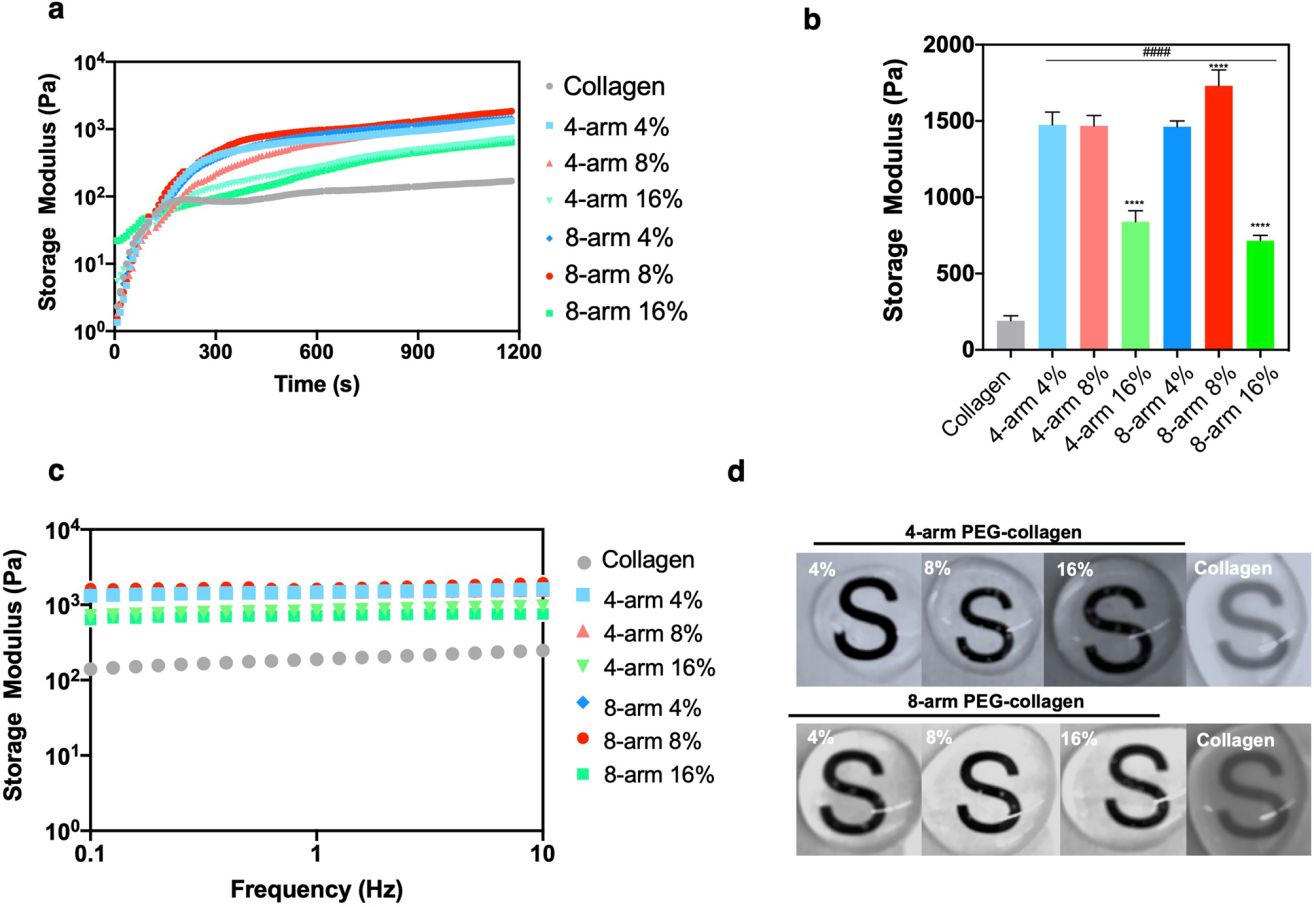
Physical properties of chemically crosslinked PEG-collagen hydrogels by NHS chemistry and non‐crosslinked (physical) collagen hydrogels. (**a**) Dynamic moduli of 4-arm PEG-collagen and 8-arm PEG-collagen hydrogels using different concentrations of PEG polymer as function of time during gelation. The hydrogels were mounted immediately after mixing. (**b**) Dynamic moduli of 4-arm PEG-collagen and 8-arm PEG-collagen hydrogels at different concentrations of PEG polymer. 4 and 8-arm PEG-collagen at 16% v/v PEG show lower storage modulus (**** p < 0.0001) compared to 4 and 8arm PEG-collagen at 4 and 8%. 8-arm PEG-collagen hydrogel at 8% showed higher storage modulus (**** p < 0.0001) compared to all other PEG concentrations and type. Collagen crosslinked with 4 and 8-arm PEG polymer at all concentrations showed significant higher storage modulus (#### p < 0.0001) compared to non-crosslinked collagen hydrogel; data is presented as mean ± SD, two-way ANOVA (p < 0.005) was used to detect statistical differences followed by Tukey’s multiple comparisons test. (**c**) Dynamic moduli of 4-arm PEG-collagen and 8-arm PEG-collagen hydrogels as a function of frequency. (**d**) Photographs of 4 and 8-arm PEG-collagen and non-crosslinked (physical) collagen hydrogels.

Here we applied NHS ester chemistry to crosslink varying concentrations of collagen using multi-arm PEG linkers in situ to generate hydrogels that can fill corneal defects and promote re-epithelialization (Fig. 1). The collagen hydrogels were crosslinked with various concentrations (4, 8 and 16% v/v PEG to collagen) of 4-arm and 8-arm PEG-NHS resulting in distinct hydrogels with different mechanical properties depending on the PEG concentration and arm number. The mechanical properties of PEG-collagen hydrogels were measured using rheological methods (Fig. 2a,b,c). The non-crosslinked collagen hydrogel reached ≈ 85 Pa in 300 s and this value increased to ≈ 141 Pa until 900 s. For the PEG-collagen hydrogel, the storage modulus was modulated by the PEG crosslinker concentration and by the PEG arm number. The storage modulus of 4% 4-arm PEG-collagen hydrogel steadily increased to ≈ 1000 Pa through 900 s. The storage modulus of 4-arm PEG-collagen with 8% PEG content was similar to that with 4%, while the 4-arm PEG-collagen with 16% PEG content had a storage modulus that remained lower on the order of ≈ 500 Pa. The storage moduli for the 8-arm PEG-collagen hydrogels with 4% PEG were very similar to those of the 4-arm PEG-collagen hydrogels. However, the 8-arm PEG-collagen with 8% PEG content reached a higher storage modulus compared to the 8-arm PEG-collagen with 4% PEG; at 900 s the storage modulus was ≈ 1320 Pa. The Fig. 2b shows that there was no statistical difference in the storage modulus of collagen crosslinked with 4 arm PEG-collagen at concentrations of 4 and 8%. Collagen crosslinked with both 4-arm and 8-arm PEG using high PEG concentration (16%) had storage moduli that were significantly smaller (****p < 0.0001) compared to those of 4 and 8 arm PEG-collagen with 4 and 8% PEG concentrations. Collagen crosslinked with both 4-arm and 8-arm PEG at all concentrations had a higher storage modulus compared to non-crosslinked collagen (####p < 0.0001). The highest storage moduli were achieved by using 8-arm PEG at 8% v/v compared to all the other groups (****p < 0.001). Thus, higher number of PEG arms increases the modulus at higher PEG concentration (up to 8%). The storage moduli of all the hydrogels at 1200 s is summarized in supplementary Table 1 and represents the moduli of the hydrogels used in the in vitro experiments. To confirm that gelation was completed, the hydrogels’ storage and loss moduli were measured as a function of frequency from 0.1 to 10 Hz (Fig. 2c,d). The moduli did not vary as a function of the frequency.

**Figure 3:**
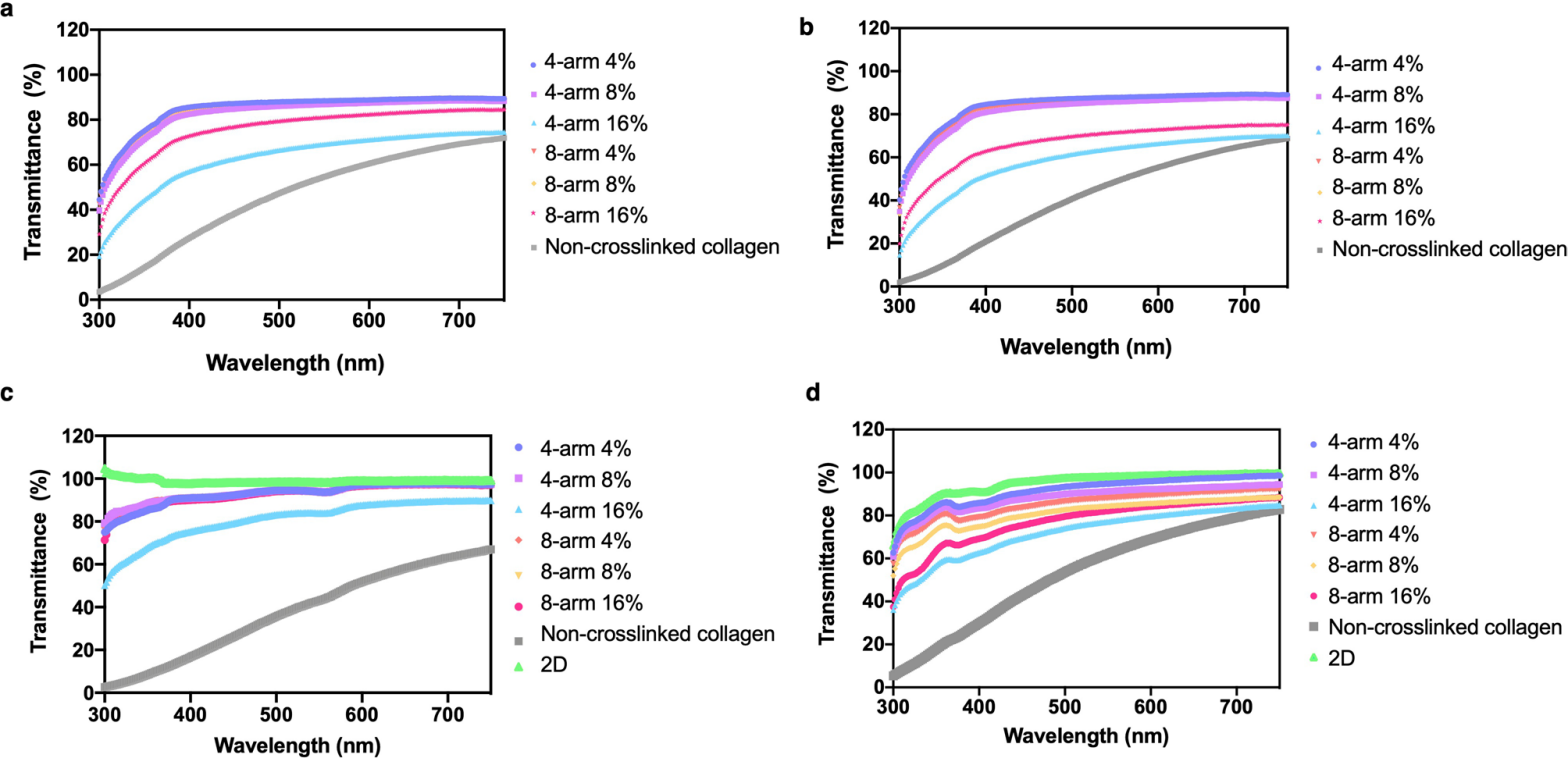
Transmittance spectra of collagen hydrogels from 350 to 800 nm (**a**) before swelling (**b**) after swelling for 24 h in PBS, (**c**) after 24 h in the presence of ICECs and (**d**) after 24 h in the presence CSSCs.

The optical properties of the 4 and 8-arm PEG-collagen and non‐crosslinked (physical) collagen hydrogels were analyzed to determine if the crosslinked hydrogels were suitable for use in the cornea. 4 and 8-arm PEG-collagen hydrogels with 4, 8 and 16% PEG content were observed to be relatively transparent while the non‐crosslinked collagen hydrogel was relatively opaque (Fig. 2d). To quantify this change in transparency, hydrogel transmittance was evaluated at wavelengths between 300 and 750 nm, before and after hydrogel swelling (Fig. 3a,b). The transmittance of 4-arm and 8-arm PEG-collagen hydrogels (with 4 and 8% PEG content) remained constant at ≈ 80% in the visible light range. The transmittance of 4-arm PEG-collagen with 16% PEG content increased with wavelength from ≈ 20% to 75%, while for the 8-arm PEG-collagen with 16% PEG increased from ≈ 30% to 80%. The non-crosslinked collagen hydrogel increased from ≈ 5% to 70%. The transmittance after swelling remained similar to that before swelling; however, for the 8-arm PEG-collagen with 16% PEG content the transparency decreased. While for other types of collagen hydrogels, swelling results in increase in transparency, this was not observed for the PEG-collagen hydrogels developed in this study^9^. In the presence of corneal cells the transmittance of the 4 and 8-arm PEG-collagen hydrogels with 16% PEG content improved compared to the hydrogels at the same concentration without the cells, after swelling (Fig. 3c,d). In the presence of human immortalized corneal epithelial cells (ICEC), the transmittance of 4-arm PEG-collagen hydrogel at 16% PEG increased from ≈ 50% to 90% and had light transmittance ≈ 80% at 500 nm, while for 8-arm PEG-collagen with 16% PEG, the light transmittance increased from ≈ 70% to 90%. Similar improvement in the transmittance was observed for the PEG-collagen hydrogels in the presence of corneal stromal stem cells (CSSC). Transmittance for both 4 and 8-arm PEG-collagen hydrogel with 16% PEG increased from ≈ 40% to 85% and showed a light transmittance of ≈ 75 at 500 nm. Bektas et al. observed that the transmittance of keratocyte-loaded 3D bioprinted GelMA hydrogels decreased compared to the hydrogels without cells, but then increased with prolonged time in culture^7^. We believe that a decrease in light transmittance in the presence of cells was not observed in this study due to extracellular matrix produced by the CSSCs and corneal crystallins produced by ICECs.^22^.

### Degradability of PEG-collagen hydrogels

**Figure 4:**
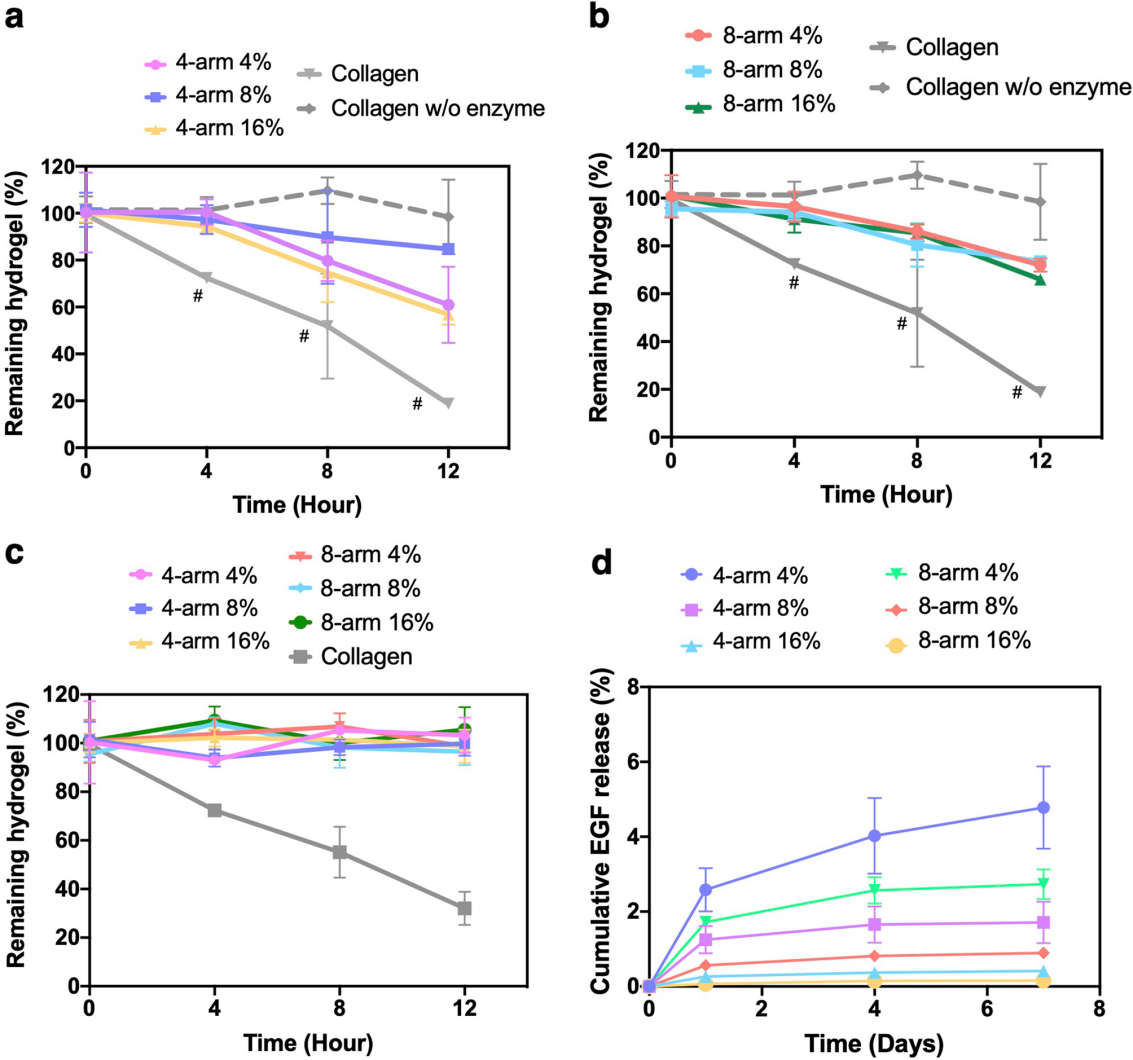
Degradation of (**a**) 4 and (**b**) 8-arm PEG-collagen hydrogels at different PEG concentrations (4%, 8%, and 16%) 24 h after swelling was evaluated in the presence of 1 mg/mL of collagenase for 12 h. Data is presented as mean ± SD, two-way ANOVA (p < 0.005) was used to detect statistical differences followed by Tukey’s multiple comparisons test. (**c**) Degradation profile of 4 and 8-arm PEG-collagen in the presence of CSSCs for 12 h. (**d**) EGF release from 4 and 8-arm PEG-collagen hydrogels during 7 days in PBS. For each condition n = 3 was used.

Hydrogel degradability is an important parameter for corneal repair and regeneration. Ideally, the hydrogel should not degrade too slowly or quickly in order to provide for proper tissue ingrowth. Here, the degradability of PEG-collagen hydrogels was evaluated after swelling in the presence of 1 mg/mL of collagenase and was monitored as a function of incubation time in complete supplemented Keratinocyte Serum Free (KSFM) medium at 37 °C (Fig. 4a,b). In the presence of collagenase, non-crosslinked collagen hydrogels degraded to about 50% of its initial mass after 8 h. After 12 h, about 20% of the hydrogel mass remained. PEG-collagen hydrogel degradation was not dependent on the number of PEG arms and concentration at 12 h. After 4 h in collagenase, the hydrogel mass was similar to time 0 for both 4-arm PEG and 8-arm PEG-collagen hydrogels. After 8 h, 20% of the PEG-collagen hydrogels was degraded, and after 12 h, 30% to 40% of the hydrogels were degraded. Overall, the mass of the hydrogels decreased with the time in collagenase. The degradation of PEG-collagen hydrogels was statistically slower compared to non-crosslinked collagen hydrogels at all evaluated times (*p* < 0.05). Of note, we chose a collagenase concentration of 1 mg/ml since it provided more than 50% degradation of the non-crosslinked collagen hydrogel. In addition, we have used the same enzyme concentration that have been used in a previous publication to degrade corneal tissue^23^. In the presence of CSSCs, PEG-collagen hydrogel degradation occurred much slower compared to the hydrogels in the presence of collagenase (Fig. 4c). At 12 h, nearly 100% of the hydrogel initial weight was still present. However, non-crosslinked collagen hydrogel degradation in the presence of cells followed a similar profile to those in the presence of collagenase. To evaluate protein release from these hydrogels, human epithelial growth factor (EGF) was entrapped in the PEG-collagen hydrogels and EGF release was evaluated over one week without collagenase. The release of EGF from the hydrogels without collagenase incubation was slow and did not significantly vary with the type and PEG concentration (Fig. 4d). After 7 days, about 95% of the EGF was still encapsulated in the PEG-collagen hydrogels.

### ICEC growth on PEG-collagen hydrogels

**Figure 5:**
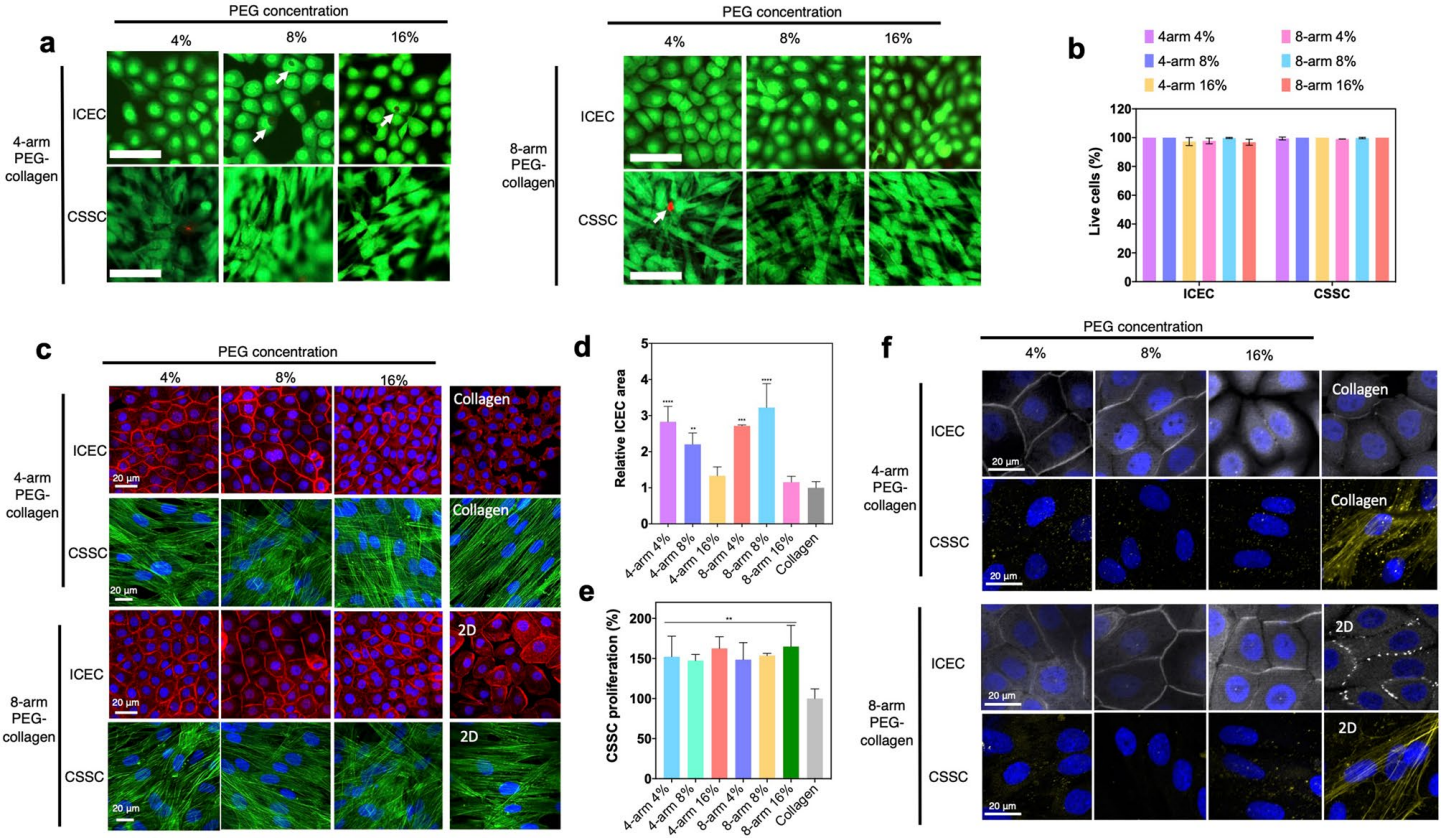
(**a**) Live/dead assays on ICEC and CSSC seeded on 4-arm PEG-collagen and 8-arm PEG-collagen hydrogels using different concentrations of PEG after 2 days in culture. Scale bar: 100 µm—live cells (green) and dead cells (red). (**b**) Viability of corneal cells seeded on PEG-collagen hydrogels. (**c**) F‐actin staining ICEC (red) and CSSC (green) showing cell morphology when seeded on 4-arm PEG-collagen and 8-arm PEG-collagen hydrogels using different concentrations of PEG after 2 days in culture. The nucleus (blue) was stained for both cells. Scale bar: 20 µm. (**d**) Relative ICEC cell area 2 days after seeding on PEG-collagen hydrogel. Areas of the cells seeded on 4 and 8-arm PEG-collagen with 4 and 8% PEG were statistically different compared to non-crosslinked collagen (****p < 0.0001, ***p = 0.0002, **p = 0.003). The data was normalized by cells seeded on non-crosslinked collagen hydrogels, with n  =  3 for each condition and ordinary one-way ANOVA applied, followed by Dunnett’s multiple comparison test. (**e**) Relative CSSC proliferation on 4-arm and 8-arm PEG-collagen hydrogels using different concentrations of PEG polymer after 2 days in culture. The data was normalized by cells seeded on non-crosslinked collagen hydrogels. A sample size of 3 and an ordinary one-way ANOVA was used followed by Dunnett’s multiple comparison test. (**p < 0.01 *vs* non crosslinked collagen). (**f**) ZO-1 (gray) staining of ICEC and ASMA staining of CSSC proliferated on PEG-collagen hydrogels for 2 days. Scale bar: 20 µm.

We next evaluated PEG-collagen hydrogels’ biocompatibility and their ability to support adhesion and proliferation of ICECs. ICEC adhesion on the hydrogels was evaluated 3 h after seeding the cells (Supplementary Fig. 1). We considered cell growth on the non-crosslinked (physically crosslinked) collagen hydrogel to be the point of comparison to calculate % cell adhesion given that it is a widely used substrate for corneal epithelial cell growth in culture. ICEC adhesion did not significantly change between the two PEG arm numbers. ICEC adhesion on 4-arm PEG-collagen did not vary as a function of PEG concentration. The same was not observed for 8-arm PEG-collagen; ICECs exhibited better adhesion at higher PEG concentration (8 and 16%) compared to 4% PEG concentration (*p* < 0.05) (Supplementary Fig. 1a). Next we evaluated cell viability in the presence of the hydrogels. ICECs were seeded on the PEG-collagen hydrogels and incubated for 2 days in complete KSFM medium at 37 °C. Material biocompatibility was then evaluated using Live/Dead assay. 4-arm and 8-arm PEG-collagen hydrogel at varying concentrations showed low or no cytotoxicity as observed by the green staining (Live cells) and very few dead cells (red staining, arrows) (Fig. 5a). Quantification of live cells showed that nearly 100% of the cells were alive after 2 days in the presence of the hydrogels (Fig. 5b). Next, cell morphology was evaluated by staining with F-actin (Fig. 5c). ICEC growth on non-crosslinked collagen hydrogels was characterized by the presence of lamellipodia and few confluent areas. ICEC behavior did not vary with PEG arm number but was observed to vary with PEG concentration. ICECs that proliferated on PEG-collagen hydrogels at 4 and 8% PEG concentration had significantly larger cell area compared to the cells that proliferated on non-crosslinked collagen hydrogels and on tissue culture polystyrene (TCP) plates (2D condition) at 2 days in culture, *p* < 0.05 (Fig. 5d). This behavior was also observed for the 8-arm PEG-collagen hydrogels. ICEC proliferation on PEG-collagen hydrogels was evaluated using MTT (Supplementary Fig. 2). ICEC grown on the non-crosslinked collagen hydrogel was considered to be 100% proliferated. ICEC seeded on 4-arm PEG-collagen hydrogels at 4 and 8% PEG concentration proliferated significantly less than they did on 8-arm PEG-collagen for both 4 and 8% of PEG concentrations, at the same time point (2 days). Interestingly, this behavior was not observed for hydrogels with 16% PEG content. Overall, cell proliferation was greater on PEG-collagen hydrogels than it was on non-crosslinked collagen hydrogels. ICEC phenotype was evaluated for the cells grown on PEG-collagen hydrogels, non-crosslinked collagen and TCP by the expression of zonula occludens (ZO-1). ICEC that proliferated on the different scaffolds expressed ZO-1 suggesting that the cells form tight junctions characteristic of a normal epithelium (Fig. 5f). Overall, the expression of ZO-1 was greater for the cells that proliferated on both 4 and 8-arm PEG-collagen hydrogels at 4 and 8% PEG content compared to the other conditions.

### CSSC growth on PEG-collagen hydrogels

CSSC behavior on PEG-collagen hydrogels was also evaluated. CSSCs seeded on 4% 4-arm PEG showed improved adhesion compared to cells seeded on 8-arm PEG hydrogel at the same concentration (^##^*p* = 0.005). For 4 arm PEG-collagen hydrogels, CSSC adhesion increased with the PEG concentration. CSSC seeded on 4 arm PEG-collagen with 16% PEG showed better adhesion compared to those with 4% PEG (*p = 0.01). CSSC adhesion on 8 arm PEG-collagen hydrogel with 4 and 16% PEG content was significantly better compared to the cells seeded on 8% (**p = 0.006 and *p = 0.01) (Supplementary Fig. 1b). CSSC seeded on non-crosslinked collagen hydrogels expressed stress actin filaments that were primarily oriented in one direction, but the same was not observed for the CSSCs seeded on the PEG-collagen hydrogels (Fig. 5c). CSSC proliferation on PEG-collagen hydrogels was evaluated using MTT (Fig. 5e). CSSC grown on the non-crosslinked collagen hydrogel was considered as the baseline control (100% proliferated). CSSC seeded on PEG-collagen hydrogels proliferated similarly at all concentrations and number of PEG arms. CSSCs seeded on non-crosslinked collagen hydrogels proliferated to a lesser degree compared to PEG-collagen hydrogels. The transformation of CSSCs into myofibroblasts was evaluated after proliferation on the different hydrogels. We evaluated the expression of alpha smooth muscle actin (ASMA), a marker of myofibroblastic activity, generally present in scar tissue or fibrosis. We observed that some cells that proliferated on non-crosslinked collagen hydrogels and TCP exhibited ASMA expression (Fig. 5f). We did not observe ASMA expression in cells that proliferated on PEG-collagen hydrogels suggesting that these scaffolds are able to preserve a more quiescent, keratocytic phenotype.

### In vivo evaluation of PEG-collagen hydrogels as fillers of corneal defects

**Figure 6:**
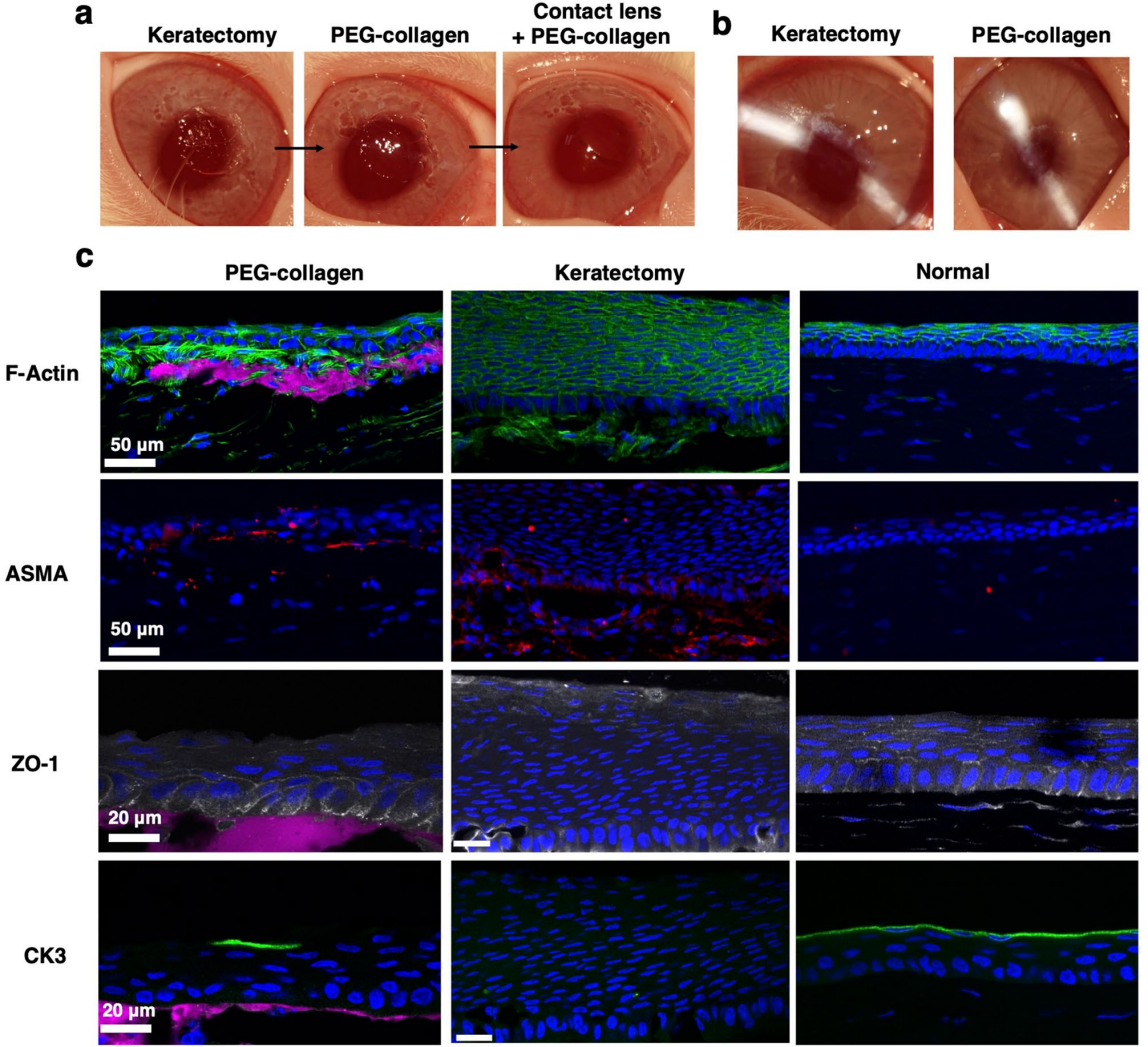
(**a**) Photographs of rabbit corneas right after keratectomy showing a rough surface and then a smooth surface after application of PEG-collagen hydrogel and contact lens. (**b**) Appearance of untreated and treated corneas on post-op day 7. (**c**) PEG-collagen hydrogel (stained magenta) underneath a multi-layered, migrated epithelial layer can be observed 7 days after treatment. For the keratectomy-only group, epithelial hyperplasia was observed. ASMA (red) was observed for the rabbits that received PEG-collagen as well as in the keratectomy group. Normal epithelial cell phenotype was detected by the presence of ZO-1 (gray) in the wing and superficial cells and CK3 (green). Minimal ZO-1 expression and no focal CK3 expression was observed for the keratectomy group.

Scaffold biointegration with host tissue is a requirement for proper tissue regeneration. Here we evaluated biointegration by the ability of the hydrogel to subsist on the stromal layer, after seven days in vivo, as well as by the phenotypic response of epithelial and stromal cells of the treated corneal tissue. Manual keratectomies were performed on the corneas of New Zealand white rabbits to simulate a stromal defect (Fig 6a, left photo). After the keratectomies were performed, the 4-arm PEG-collagen hydrogels with 8% PEG content were applied to the defect sites (Fig 6a, middle photo) and then bandage contact lenses were placed on the eyes to protect the gel-treated cornea, followed by tarsorraphy (sutured eyelid closure). After 1 week, PEG-collagen hydrogels were able to support the growth and migration of epithelial and stromal cells (Fig. 6c, first column). Three rabbits were tested (n = 3). We selected the 4-arm PEG-collagen with 8% PEG on the basis of the fact that it exhibited the greatest transparency (Fig. 3) in the presence and absence of cells compared to all other formulations, in the context of the fact that it also compared well to other formulations in all other biophysical experiments. The impact of using the 8-arm PEG-collagen as an ocular adhesive to sustain cell migration and wound healing was also studied in one rabbit with similar results shown in Supplementary Fig. 4. Figure 6a shows that after the application of the hydrogel, the stromal defect becomes smooth compared to the rough surface right after keratectomy. Slit lamp exam on post-operative day 7 revealed that there were no clinical signs of anterior segment inflammation after treatment (Fig. 6b). One week after surgery, the animals were euthanized and the corneas were analyzed for the migration and proliferation of epithelial cells over the hydrogels, presence of ASMA, ZO-1 and CK3 (Fig. 6c). We found that the PEG-collagen hydrogels were able to support the growth and migration of epithelial cells and showed good apposition to the underlying stromal bed (Fig. 6c). Numerous epithelial layers strongly marked by F-actin staining were observed in the no treatment group indicative of epithelial hyperplasia (Fig. 6c). This was not observed in the groups that received the hydrogel treatment. Myofibroblasts were found to be present in both the treated and control corneas 7 days after the keratectomy. ZO-1 staining was observed in the epithelial superficial layer and wing cells of the treatment group after keratectomy (Fig. 6c). The control group showed ZO-1 staining in the most-superficial cells but not in the underlying wing cells. Epithelial cells that migrated over the hydrogel were able to express CK3 in some areas showing that the cells are differentiating into mature corneal epithelial cells. Corneas from the control group exhibited no focal expression of CK3, suggesting abnormal phenotype. In addition, 8-arm PEG-collagen hydrogel (8%) was also able to support epithelial cell migration and phenotype while few ASMA positive cells were observed (Supplementary Fig. 4). In summary, PEG-collagen hydrogels were able to fill the defect area and remained transparent over one week and supported multi-layered epithelial growth along with markers of normal differentiation (ZO-1 and CK3).

## Discussion

PEG-based ocular adhesives have been approved by the FDA to seal clear corneal incisions commonly used in cataract surgery^24^. In addition to PEG-based materials, currently available corneal adhesives are based on biological fibrin or synthetic cyanoacrylate glues, although both of these are used off-label. The main goal of cyanoacrylate, for instance, is to prevent corneal perforation and to serve as a temporizing measure before a corneal transplant^25^. Despite their nontoxicity and low immunogenicity, these material have some limitations with regard to stability and gelation time^4^ and, in the case of cyanoacrylate, a complete lack of optical transparency or biointegration. Alternative treatments for stromal defects are being explored that improve patient outcomes—since there are no technologies specifically approved for the filling and regeneration of stromal defects. Collagen, hyaluronic acid and gelatin based fillers are being investigated as alternatives to the commercially available treatments to provide better biointegration with host tissue^3,8,26^. These corneal fillers should be degradable and support corneal cell migration and proliferation.

In prior work, we developed in situ-forming collagen hydrogels crosslinked by strain-promoted azide-alkyne cycloaddition (SPAAC) using bovine collagen type 1. These hydrogels were able to sustain the growth of both human keratinocytes and keratocytes^12^. Here we have developed in situ forming collagen hydrogels crosslinked by multi-functional PEG-N-hydroxysuccinimide that exhibited suitable mechanical and biological properties to fill and repair corneal stromal defects. PEG-collagen hydrogels have been previously evaluated for cytocompatibility, and fibroblast proliferation and migration^17^. In our system, bovine collagen type 1 (3 mg/mL) was crosslinked with multi-arm PEG-NHS via primary amine ester reaction. These hydrogels are formed in situ and are transparent and biocompatible. Our results showed that these hydrogels’ mechanical properties can be tuned by varying PEG concentration and arm number. In addition, PEG concentration and arm number had effects on transparency, storage modulus and degradation profile. High concentration of PEG negatively impacted the gels’ biophysical properties. For instance, the transparency and the storage modulus decreased at 16% PEG content. We believe that there was a saturation effect, where beyond a certain concentration of PEG, no further crosslinking was achieved, and any additional PEG served only to reduce crosslinking density and also decreased transparency via inadequate macromolecular mixing leading to matrix heterogeneity. At lower concentrations, crosslinking and mixing are well-balanced, leading to more transparent hydrogels.

Both PEG and collagen have been explored in tissue engineering due to their biocompatibility and ability to be easily modified to create in situ scaffolds with different mechanical properties^17,27^. These properties are particularly attractive for corneal regeneration applications that require safe and transparent scaffolds^28^. We showed that corneal epithelial and stromal cells were able to proliferate, adhere and had desirable morphology when grown on the PEG-collagen scaffolds. ICECs proliferate to a greater degree on PEG-collagen hydrogels versus non-crosslinked collagen and were able to form a monolayer. CSSCs did not vary between the PEG-collagen hydrogels in terms of morphology but were able to proliferate more effectively on crosslinked collagen hydrogels than on non-crosslinked collagen. The number of PEG arms but not the storage modulus impacted corneal cell behavior. The different behaviors observed for corneal cells on crosslinked and non-crosslinked collagen was previously investigated to be related to the alignment of the collagen fibers as a result of PEG crosslinking^29^. Here we chose to move forward with the 4-arm PEG-collagen formulation with 8% PEG for the in vivo studies due to its superior transparency in the presence or absence of cells (Fig. 3) and the blend of desirable mechanical properties and cytocompatibility found through our in vitro experiments. The 8-arm PEG-collagen at 8% PEG content did show promising wound healing results similar to those of the 4-arm gel, but requires further evaluation with additional animals in future studies.

In vivo experiments revealed that the in situ forming PEG-collagen hydrogel was effective as a corneal defect filler that supported re-epithelialization in vivo one week after application. These hydrogels were likely bound to the stromal wound bed to some extent due to reactivity of NHS moieties with primary amines in stromal collagen^17^ given that the same chemistry is used to seal corneal incisions^21^. Of note, epithelial hyperplasia was observed in the keratectomy-only group and not the PEG-collagen treatment group, a phenomenon that is a function of stromal defect depth^30^. The presence of PEG-collagen hydrogels provided an exogenous stromal matrix on the wound bed that supported multi-layered epithelial migration and proliferation as well as ZO-1 and CK3 expression^31^. In normal corneal stromal wound healing, corneal fibroblast infiltration followed by conversion to myofibroblasts is observed^32^. In our study, myofibroblastic responses were present in the cornea of animals that received the PEG-collagen gel and in the untreated corneas as expected after stromal injury, although to a qualitatively lesser extent in the treated corneas.

In summary, we have shown that collagen hydrogels crosslinked via multi-arm PEG succinimidyl esters can be formed rapidly in situ and can support multi-layered re-epithelialization when applied to corneal stromal defects. The hydrogels form rapidly under ambient conditions without the need for a chemical or photochemical trigger. The material represents a promising in situ stabilizer of corneal defects that, in contrast to cyanoacrylate glue, has the potential to maintain vision due to its transparency and promote healing by supporting surface epithelialization. Although they were found to degrade in the presence of collagenase in vitro, limited degradation was seen in vivo but is certain to occur over time, and thus longer follow up intervals are needed to understand what affect this has on the treated corneas in terms of transparency and anatomic outcomes. Additional studies that evaluate longer follow up periods, different wound diameters and depths, and additional formulation variations are merited to further understand the therapeutic potential of this material in the repair of corneal wounds.

## Methods

### Fabrication of PEG-collagen and non-crosslinked collagen hydrogels

Type I bovine collagen (Thermo Fisher Scientific; A1064401) was first pH neutralized using a solution of 1.0 M sodium hydroxide solution, deionized (DI) water, and 10 × PBS in a 3:57:20 ratio. The 5 mg/mL collagen solution was mixed with the neutralization solution in 3:2 ratio so that the final concentration of collagen was 3 mg/mL. Neutralized collagen was conjugated to 4 or 8 arm PEG Succinimidyl NHS ester 10 K (Creative PEGworks; PSB-4413 and PSB-846) via NHS chemistry to react with collagen's primary amines. First, PEG was solubilized in PBS to give a concentration of 100 mg/mL, then 4, 8 and 16 μL of this solution was added to 100 μL of neutralized collagen. Next, the mixed solution of PEG and collagen were put at 37 °C for 30 min to ensure gelation of the hydrogels. For non‐crosslinked collagen hydrogels, the collagen was neutralized as we mentioned above and incubated for 30 min at 37 °C according to the collagen gelation procedure of Thermo Fisher Scientific. The non-crosslinked collagen is a physical crosslinked hydrogel formed by the neutralization of collagen at 37 °C. Although it is not a covalently crosslinked system, it does form a physically crosslinked gel that is relatively strong and allow cell studies in these systems over several days^12,33^. To visualize the hydrogel for the in vivo experiments, Alexa Fluor 647 NHS Ester (Thermo Fisher Scientific, A20006) was conjugated to the neutralized collagen following the manufacturer’s instructions. Briefly, the collagen conjugated to Alexa 647 was incubated for 2 h at 4 °C and dialyzed via Slide-A-Lyzer dialysis kit (Thermo Fisher Scientific, 66,382) overnight at 4 °C in PBS. Conjugated collagen 647 (50 μL) was mixed with neutralized collagen (100 μL) before adding the PEG-NHS reagent. Aseptic procedures were used at all times. For the in vitro and in vivo experiments, a bottle of sterile collagen type 1 bovine was opened under the hood where neutralized collagen and PEG was mixed together to form hydrogels as described above. For the in vivo studies, the PEG and collagen reagents were prepared under the hood and only opened at the time of surgery on a sterile field and handled using sterile instruments.

### Mechanical characterization of PEG-collagen hydrogel

The mechanical properties of the collagen hydrogels were evaluated using an ARES‐G2 rheometer (TA Instruments, New Castle, DE, USA) at Stanford Soft & Hybrid Materials Facility (SMF, Stanford, CA, USA). For the non‐crosslinked collagen hydrogel, neutralized collagen was mounted on the plate and measured. For the PEG-collagen hydrogels 4 or 8-arm PEG NHS solution was added to the neutralized collagen solution. Then the 150 μl of mixed solution was mounted on the plate immediately after mixing with a pipette. To determine gelation time, time sweeps were performed at 37 °C for 1200 s at 1% strain and 1 Hz oscillatory frequency. Next, frequency sweeps from 0.1 to 10 Hz with a fixed 1% strain were performed to determine the completion of gelation. To evaluate how the mechanical properties of the hydrogels could be modulated, PEG-collagen hydrogels were formed at different concentrations 4, 8 and 16% of PEG to collagen. To examine the mechanical properties at complete gelation, the resultant solutions were deposited onto a glass substrate and incubated at 37 °C for 2 h, and then frequency sweeps from 0.1 to 10 Hz with a fixed 1% strain were performed. The storage moduli was found to nearly equivalent to those found at 1200 s in Fig. 2a.

### PEG-collagen hydrogel transparency evaluation

The hydrogels' absorbance from 350 to 800 nm was measured using a SpectraMax M Series Multi‐Mode Microplate Reader before and 24 h after swelling in 100 µL of PBS. The PEG-collagen and non‐crosslinked collagen hydrogels were fabricated in a 96 well plate, and the volume was 100 µL. The hydrogel transmittance was also evaluated at 24 h in the presence of the cornea cells. Briefly, 1 × 10^5^ cells/mL were seeded on the hydrogels in a 96 well plate and 24 h later, the medium was removed and loose cells were washed out with PBS before reding the hydrogel’s absorbance. The absorbance was converted to transmittance using the relation A = 2 − log10 (%T). For each condition, n = 3 was used and the absorbance of the medium without phenol red was subtracted from the hydrogel’s absorbance before converting to transmittance.

### Multi-arm PEG-collagen hydrogel degradation

We used two methods for evaluating the degradation of 4 and 8-arm PEG-collagen hydrogels with 4%, 8%, and 16% PEG content and non-crosslinked collagen hydrogels. Degradation was studied in the presence of collagenase or in the presence of seeded CSSC cells. Eppendorf tubes were weighed and 70 µL of each hydrogel was synthesized inside of the tubes. Next, 140 µL of 1 mg/mL of Collagenase from Clostridium histolyticum type I, 0.25–1.0 FALGPA units/mg solid, ≥ 125 CDU/mg (Sigma Life Sciences; C0130) in warm supplemented Keratinocyte Serum Free Medium—KSFM (Thermo Fisher Scientific; 17,005,042) containing Bovine Pituitary Extract (BPE), EGF, 100 ng/ mL of hydrocortisone (Sigma Aldrich; H0008) and 5 µg/mL of insulin (Sigma Aldrich; 0516) was added to the tubes containing the hydrogel. Untreated hydrogels received 140 µL of warm KSFM with no collagenase. The tubes were placed on a platform rocker, allowing the collagenase to react with the hydrogel. For hydrogel degradation in the presence of seeded CSSC cells, 1 × 10^5^ cells/mL were added to the swollen hydrogels in cell culture media. After 0, 4, 8, and 12 h, the supernatant was removed, leaving the remaining hydrogel behind (with or without cells). At each time the tubes were weighed and subtracted from the original weight of the tube with hydrogel (time 0) to obtain the wet weight of the hydrogel. For each condition n = 3 was used. The hydrogel weight at time 0 was considered 100% of the hydrogel weight. The degradation of the hydrogels was compared to the degradation of non-crosslinked collagen hydrogel with and without collagenase or cells (control).

### Epidermal growth factor release

The EGF release was evaluated as previously described^34,35^. Briefly, the PEG-collagen hydrogel including EGF was fabricated in 48-well plates. The initial volume of solution was 100 µl. After mixing neutralized collagen with 4 or 8 arm PEG-NHS solution in each well, the hydrogel was incubated at 37 ºC for 30 min. At each well, 500 µl of PBS solution was applied, and the solution was collected and refreshed at each time point. The amount of released EGF was measured by EGF ELISA kit (30X wash buffer, biotinylated antibody reagent, streptavidin-HRP concentrate, TMB substrate, and stop solution containing 0.16 M sulfuric acid; Thermo Fisher Scientific, Waltham, MA, USA), and we followed the EGF ELISA kit protocol. Briefly, the collected solutions were added to the capture antibody-coated well plate and incubated at 37 ºC for 2 h. The biotinylated antibody reagent and diluted streptavidin-HRP in PBS were added to the well and incubated for 2 and 1 h in turn, respectively. After each step, all wells were washed three times using the washing buffer in the kit. For the color development, TMB solution was added and incubated in the dark for 30 min at room temperature, and stop solution was added to stop the reaction without washing. The absorbance of each resultant well was measured at 550 nm using SpectraMax M Series Multi-Mode Microplate Reader (Sunnyvale, CA, USA). For quantification of immobilized EGF, a standard curve of EGF ELISA was obtained following the established protocol of instruction, and the amount of EGF was calculated. The total amount of added EGF was calculated, and the released amount of EGF was divided by the total amount of initial EGF.

### Corneal epithelial cell culture

ICEC were kindly donated by Dr. Djalilian’s laboratory from the University of Chicago, Illinois. ICECs were culture in supplemented KSFM. After the cells reached 80% confluency, they were passaged. The medium was changed every other day.

### Corneal stromal stem cell culture

CSSC were harvested from human donor corneas provided by Lions Eye Institute. Research corneas were in Optisol GS had minimum cell count of 2000, maximum death to preservation time of 7 days, and no history of HSV/VZV/HIV/Hepatitis, age ranging from 30–35 years old. First, the endothelial layer was removed, as well as the central corneal using a trephine (8 mm). The remaining limbal region was dissected into small fragments and placed epithelial side down on 6 well plates. CSSCs were given time to transplant onto a flask bottom over a period of 7 days in Minimum Essential Medium Eagle (Sigma Aldrich; M4526) containing 10% Fetal Bovine Serum (FBS), 1% antibiotic antimycotic solution (Sigma Aldrich; A5955), 1% Non-essential Amino Acid Solution (Sigma Aldrich; M7145) and 1% Glutamax (Thermo Fisher Scientific; 35,050,061). After reaching 80% confluence, the CSSC culture was subcultured into colonies and used until passage 4.

### Corneal cell biocompatibility, morphology and proliferation on multi-arm PEG-collagen hydrogels

Cornea cell viability was evaluated using Live/Dead assay according to Thermo Fisher Scientific protocol. Briefly, 200 μL of PEG-collagen hydrogels were formed within a 48 well plate as previously mentioned. Complete gelation of the hydrogel was achieved by incubating the hydrogels at 37 °C for 20 to 30 min.”. Next, supplemented KSFM was added to the hydrogels and left overnight. Next, 2 × 10^5^ cells were seeded on the hydrogels for two days. Live/dead solution was added, and the cells were evaluated under inverted microscopic. Cellular morphological behavior of ICEC and CSSC were evaluated by staining the cells with Alexa Fluor 488 Phalloidin (Thermo Fisher Scientific; A12379) and Rhodamine Phalloidin (Thermo Fisher Scientific; R415). Phenotype evaluation was assessed by staining the cells with ZO-1(Thermo Fisher Scientific; ZO1-1A12) and ASMA (Abcam; ab5694). First, the hydrogels (130 μL) were formed in an 8 well chamber slide and 5 × 10^4^ cells were seeded on the hydrogel. After 2 days, the cells were fixed with 4% paraformaldehyde (PFA) for 15 min, permeabilized and blocked with 0.5% triton-X and 5% goat serum (GS) in PBS for 30 min. Primary antibodies in permeabilizing and blocking solution were added and incubated overnight at 4 °C. In the next day, secondary antibody Alexa Fluor 488 in the same antibody solution was added for 2 h. Then Alexa Fluor 488 Phalloidin or Rhodamine Phalloidin were added to the cells in PBS (1:40) for 30 min. Next, 4′,6-diamidino-2-phenylindole (DAPI) was added in PBS for 5 min. The cells were then analyzed using confocal microscopy. Cell proliferation was evaluated using Thiazolyl Blue Tetrazolium Bromide (MTT, Sigma Aldrich; M5655). Briefly, the cells 5 × 10^4^ were seeded on PEG-collagen hydrogels formed in 96 well plates. After 2 days, the MTT solution was added for 2 h. The crystals were solubilized using Dimethyl Sulfoxide (DMSO). The absorbance was read at 570 nm. Corneal cells plated on the wells without hydrogels, were considered to have 100% of proliferation.

### In vivo keratectomy studies in rabbits

Adult New Zealand white rabbits were used to conduct lamellar keratectomy studies. Animal experiments were designed to conform with the ARVO statement for the Use of Animals in Ophthalmic and Vision Research and were reviewed and approved by the Stanford University Institutional Animal Care and Use Committee. All anesthesia techniques were performed by the veterinary service center (VSC) at Stanford University. Prior to surgery, one drop of proparacaine hydrochloride ophthalmic solution was added to the eye receiving treatment. A manual lamellar keratectomy was performed on the right eye using a 3.5 mm customized vacuum trephine to create a deep circular cut and a spatula was used to remove the anterior stroma. Approximately 5 µL of 4-arm PEG-collagen-Alexa 647 hydrogels at 8% PEG concentration were applied to the keratectomy area and allowed to gel in situ for 1 min before applying the contact lens and performing a tarsorrhaphy. The corneas from the 4-arm PEG-collagen at 8% group (n = 3) were compared to the keratectomy group (n = 3, control) clinically for signs of inflammation or infection. The 8 arm PEG-collagen was also applied to the keratectomy area of a rabbit cornea (n = 1). Ofloxacin ophthalmic solution was applied daily. On day 7, the tarsorrhaphy and the contact lens were removed for clinical examination under a surgical microscope and photographs using a Paxos Scope smartphone-based ophthalmic camera adapter. On day 7, the rabbits’ eyes were enucleated for immunohistochemical processing and evaluation.

### Immunohistochemistry

The tissues were sectioned, and cross‐sections of corneal tissues were fixed with PFA 4%, permeabilized and blocked as mentioned above. Primary antibodies ASMA, ZO-1 and CK-3 (Abcam; ab77869) were incubated in 0.5% triton-x and 5% NGS overnight. Next the secondary antibody anti-mouse Alexa 488 for CK3 was added for 2 h. Finally, F-actin and DAPI were added for 30 and 5 min, respectively. The corneas cross‐sections were analyzed under confocal microscopy.

### Statistical analysis

All data are expressed as the mean ± standard deviation (SD). Each experiment was repeated at least 3 times unless otherwise indicated. Statistical evaluation performed are informed on each Figure. A value of *p* < 0.05 was considered statistically significant. The statistical analysis was performed by using GraphPad Prism 7 statistical software^35^.

## Supplementary information

Supplementary file1

## Data Availability

The analyzed datasets from this study are available from the corresponding author upon request.
