# Supplementary material for: In situ-forming collagen hydrogel crosslinked via multi-functional PEG as a matrix therapy for corneal defects
Source: Sci Rep. 2020 Oct 7;10:16671. doi: 10.1038/s41598-020-72978-5 (PMC7542443; doi:10.1038/s41598-020-72978-5)
Supplement: Supplementary file 1 — Supplementary file1 [file 41598_2020_72978_MOESM1_ESM.pdf]

Supplementary Materials for:

In situ-forming collagen hydrogel crosslinked via multi-functional PEG as a matrix therapy for corneal defects

Table S1: PEG-collagen storage modulus values at 1200 seconds at different PEG concentration and arm numbers.

	**Storage modulus at 1200 seconds (Pa) ± standard deviation (SD) of different v/v PEG to collagen solution**			
**Number of PEG arms**	4%	8%	16%	Collagen
4	1327.4 ± 21.8	1339.7 ± 18.7	721.2 ± 12.3	169.9 ± 1.7
8	1455.6 ± 7.8	1842.2 ± 30.5	639.6 ± 9.8	

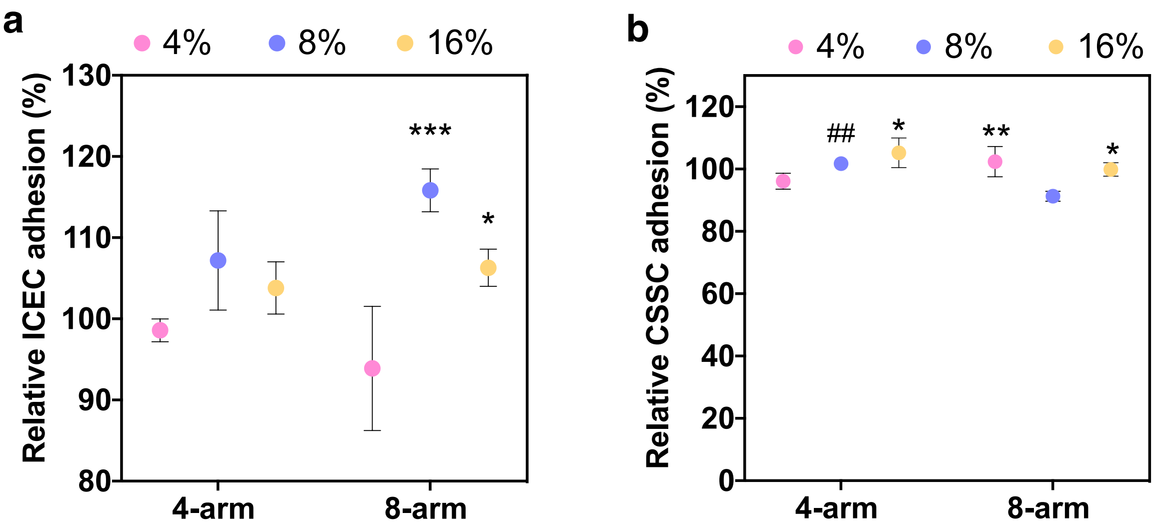

Figure S1. a. Relative ICEC and **b.** CSSC adhesion on 4 and 8-arm PEG-collagen hydrogels 3 hours after seeding, normalized to ICEC and CSSC cell adhesion on non-crosslinked collagen hydrogels, respectively. ICEC adhesion on 8-arm PEG-collagen with 8% and 16% PEG concentration was statistically better (* p = 0.01, *** p = 0.0002) compared to those with 4 % PEG. CSSC seeded on 4-arm PEG-collagen with 8% PEG content exhibited better adhesion compared to 8-arm PEG-collagen at the same concentration (## p = 0.005). CSSC exhibited greater relative cell adhesion on 16 % 4-arm PEG-collagen hydrogel (* p =0.01) compared to the other

concentration at same arm number. CSSC exhibited greater relative cell adhesion on 8-arm PEG-collagen with 4 % and 16 % PEG content compared to those with 4 % (* p = 0.01 ,** p = 0.006). Data is presented as mean ± SD, two-way ANOVA ($p < 0.005$) was used to detect statistical differences followed by Tukey's or Sidak's multiple comparison test.

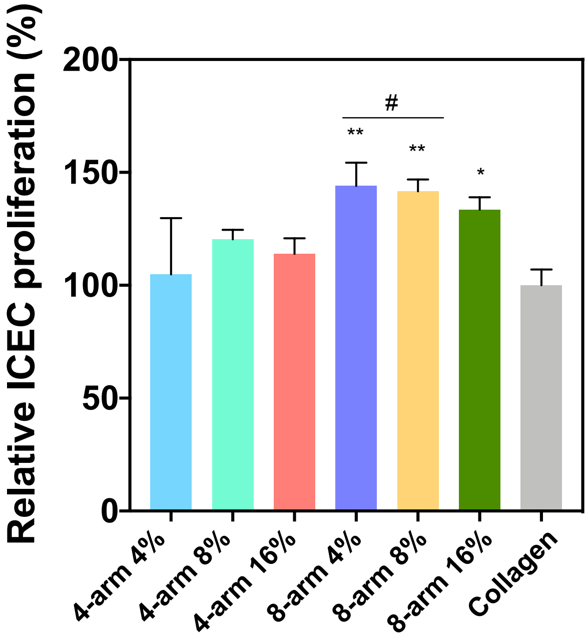

Figure S2. Relative ICEC Proliferation on 4-arm PEG-collagen and 8-arm PEG-collagen hydrogels normalized to cell proliferation on non-crosslinked collagen hydrogels using different concentrations of PEG polymer after 2 days in culture (*p = 0.01, ** p =0.02 *vs* non crosslinked collagen). Sample = 3 and Ordinary One-way ANOVA was used followed by Dunnett's multiple comparison test.

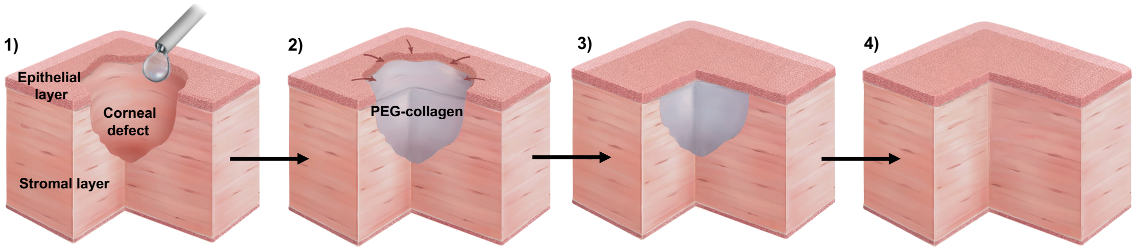

Figure S3. Schematic of corneal defect and repair by in situ PEG-collagen hydrogel treatment.

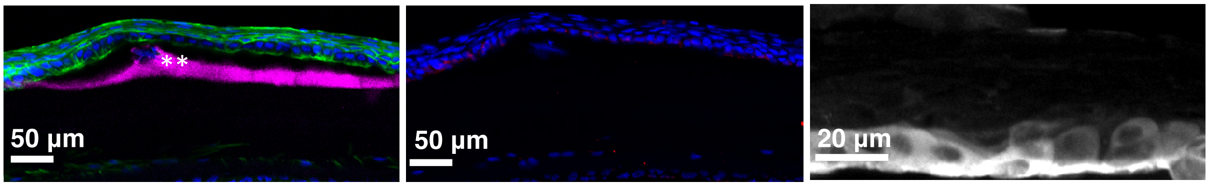

Figure S4. 8-arm PEG-collagen hydrogel with 8% PEG content (stained magenta, asterisks) underneath a multi-layered, migrated epithelial layer (stained green) can be observed 7 days after treatment. Few ASMA positive cells (red) was observed for the rabbit that received PEG-collagen. Normal epithelial cell phenotype was detected by the presence of ZO-1 (gray).
